# “Seeing Color,” A Discussion of the Implications and Applications of Race in the Field of Neuroscience

**DOI:** 10.3389/fnhum.2019.00280

**Published:** 2019-08-13

**Authors:** Sade J. Abiodun

**Affiliations:** ^1^Duke Institute for Brain Sciences, Duke University, Durham, NC, United States; ^2^Center for Cognitive Neuroscience, Duke University, Durham, NC, United States

**Keywords:** diversity, race, representation, racism, Bias

Between 1986 and 2006, the number of North American institutions offering undergraduate degrees in Neuroscience increased 20-fold. This boost mirrored the modern wave of Neuroscience, dubbed by the United States Congress as the “Decade of the Brain.”

However, the true emergence of the discipline of brain science dates long before the 2000s, finding its roots in the theorems and postulates of some of the most celebrated minds in the fields of Psychology, Biology, and related disciplines. Neuroscience has established its presence as a strong force in the scientific community, far surpassing the shelf life characteristic of a common pseudoscience, but some say the field has yet to undergo the meticulous scrutiny that more long-standing disciplines have endured; an evaluative process which includes a review of the ethical implications and impact of the field itself.

With the ever-progressing code of ethics and morality in modern society, one integral component has been the discussion of the inclusion and representation of marginalized identities within the scientific community, and how—for better or for worse—scientific disciplines have evolved from their original culture of the perennial “old boys club,” welcoming only the most racially and historically privileged individuals in society. The question that this brings up, that this commentary is designed to tackle, is whether these same discussions around diversity in the wider realm of science have happened and should happen in the field of Neuroscience.

By examining both the historical and present-day dynamics of this field, this manuscript examines whether the Neuroscience community has successfully been held accountable for its actions, or whether the attempt to remain “objective” has, in essence, resulted in harmful complicity in the perpetuation of scientific racism.

## In the History Books

Many of the modern-day discoveries within the fields of Psychology and Neuroscience still pay homage to the methodologies and theorems developed decades before. This reverence, however, brings into question the extent to which the glorification of the historical figures behind these theories conveniently overlooks some of their more problematic beliefs.

In the late nineteenth century, Dutch and German scientific duo Gustav Fritsch and Eduard Hitzig gained recognition for their behavioral research that led to a deeper understanding of the motor cortex and basal ganglia. Fritsch and Hitzig are canonically lauded as scientific pioneers for their contributions to the model of motor function. It is a lesser known fact, however, that Fritsch's anthropological explorations and research were motivated by his quest to find proof of the genetic superiority of the white race. Fritsch's ethnographic work allowed him to travel to countries around Europe and Southern Africa, where he spent months at a time collecting eye and hair evidence aimed at finding an anthropological basis for the genetic inferiorities of “negroes” (Gross, 2012). This erasure is not restricted to a single scenario: upon further research, it becomes clear that even a handful of names one might find in a History of Neuroscience syllabus had more than a few, often lesser-known controversial ideological stances. David Hume, Scottish philosopher and contributor to *Discourse on Brain and Consciousness*, stated “I am apt to suspect the negroes, and in general all the other species of men to be naturally inferior to the whites. There never was a civilized nation of any other complexion than white, nor even any individual eminent either in action or speculation” (West, 2003). Immanuel Kant, metaphysicist and inspiration behind Kantian Neuroscience, once said, “Among the hundreds of thousands of blacks who are transported elsewhere from their countries… still not a single one was ever found who presented anything great in art or science or any other praiseworthy quality, even though among the whites some continually rise aloft from the lowest rabble, and through superior gifts earn respect in the world” (West, 2003). And Voltaire, French Enlightenment philosopher and influencer of *Theory of Mind*, wrote “The Negro race is a species of men as different from ours as the breed of spaniels is from that of greyhounds,” in his essay “The People of America” (West, 2003).

Students are often taught about the integral contributions of these scientists, including their experiments, methods, and even some of their *academically* contentious stances. But little to no light is cast on their ideological viewpoints, some of which served as key motivators and components of their overall bodies of academic work.

But where do we draw the line between brilliance and ignorance?

It is undeniable that, in many ways, these scientists have been the backbone of Modern Neuroscience, individuals whose knowledge and prestige precede them. However, we cannot solely acknowledge the positive and/or beneficial aspects of their legacy, but should also recognize the importance of identifying the successes *and* shortcomings which, together, make up their bodies of belief. This legacy of accurate and holistic representation becomes all the more important when considering how race and ethnic diversity is represented within Neuroscience research itself.

## Neuroscience of Race

In recent years, many of the Natural Sciences have delved into the practice of investigating “social” issues from an empirical perspective. This discipline, dubbed “cultural neuroscience,” has examined behavioral phenomena related to prejudice, social segregation and the like, to help us understand how our attitudes form (Choudhury and Kirmayer, 2011; Martínez et al., 2012). As highlighted in some of the systematic reviews done on current literature in the field, these studies have outlined the behavioral schema by which we associate and subsequently react to variations in race, gender, and other phenotypic characteristics (Ronquillo et al., 2007; Richeson et al., 2008; Martínez et al., 2012). Such research has confirmed that factors such as Cross-Race Effect and Implicit Bias can explain the unconscious assumptions we make about different groups, and neurological responses that occur when confronted with people different from ourselves (Phelps et al., 2003; Wheeler and Fiske, 2005; Kubota et al., 2012). Applying these findings to our understanding of everyday social encounters, this research has provided objective evidence challenging the preconceived notion of racism as a fictitious experience.

However, this empirical approach could lead to the pathologization of racism, presenting it as a clinical matter of evolutionary advantage rather than as problematic behavior (Rose and Rose, 2001; Martínez et al., 2012). By depicting of a man's hatred for his immigrant neighbor as a specified pattern of activation in fusiform face area and amygdala rather than an act of ignorance and hatred, we create a much more docile interpretation of the situation, which negates and justifies the potential violence of one human's emotions toward another.

Additionally, the attribution of cultural difference to physical distinction is the very belief from which early scientific practices such as Phrenology, Physiognomy and Trepanation found their footing. Phrenology, a field created by Franz Joseph Gall in the late eighteenth century, was centered around the idea that the brain could be divided into an aggregated map, topographically organized into characteristics and traits. With this ideology came many levels of prejudicial assumptions on the mental capacities and differences of various ethnic and racial groups, with whiteness often being centered as the “pinnacle specimen of intelligence” (Gross, 2012; Staum, 2014). Similar racial iterations propagated the popularity of scientific documents such as *Crania Americana*, which many justified the implementation of the Trans-Atlantic Slave Trade and labor exploitation of black and brown bodies.

Although this same line of reasoning may not be explicitly employed today, a key element for the “weaponization” of the scientific findings of that age was the lack of accountability on the part of the scientist for the implications of their research. The conclusions drawn at a lab bench or computer desk—a controlled environment in which the members of that cohort adhere to a certain united ethical code—does not always similarly translate when published to the larger population. These scientific ideas and conclusions can be warped and manipulated, and in some cases, even used to push dangerous agendas of racial and cultural hierarchy. This is one benefit of having diverse perspectives within the research community, which facilitates consideration of the overarching influence of research, and examination of whether marginalized voices have been given a space within field's framework.

## Neuroscience and Race

While it is important to talk about the applications of race within the data, it is also critical that we evaluate the application of racial literacy within the Neuroscience community. Just as the brain is composed of bundles of individual neurons and synapses, a scientific field is equal to the sum of its parts: principal investigators, research assistants and technicians, post-doctoral scholars, and undergraduates.

Statistically speaking, Underrepresented Minorities (URMs) comprise only 7.7% of the larger scientific community in the United States of America (Higher Education Training., 2013), with an even smaller percentage of this subpopulation (<2%) counting black and Hispanic women (Matchett, 2013; Guterl, 2014). But even these numbers are a far cry from the minuscule demographic representation of the past. In a 1974 governmental study, it was found that 94% of neuroscientists were white, with a mere 1.8% representing the cumulative total of all URM's in the field (2011, Higher Education Training., 2013). As it stands today, Neuroscience appears (on paper) to still be a very white, male, western-dominated field: within academia, URMs represent only 12% of pre-doctoral students, 4% of postdocs, and 6% of all tenure-track faculty across Neuroscience departments nationwide (Higher Education Training., 2013). While these numbers are shocking, they are not surprising considering the historical landscape of access and availability. A little over 3 decades ago the Society for Neuroscience (SfN), the largest Neuroscience society in the world, had <4% of their entire member body identify as racial minorities, a fact that incited the development of diversity initiatives such as the Neuroscience Scholars Program (NSP) in an effort to bolster recruitment (Matchett, 2013; Research Funding, 2018). Even with these initiatives and platforms, however, the numbers show that significant work is still to be done before the playing field is level for all.

The more prominent instances of minority representation in research are not often from the investigator's side, but rather in the populations utilized to conduct the research. Both early and modern Neuroscience, like many other research fields, have made use of the most “available” subject populations to collect data, which usually entailed exploiting lower socioeconomic status (SES) communities and People of Color (POC's), who were often less protected and more at risk. In the past, this has included the use of groups categorized as “vulnerable populations,” including prisoners, mentally unstable patients, and other marginalized groups (pregnant women, fetuses, institutionalized patients, etc.) (Fiscella et al., 2000; Backlar, 2002). Many of these vulnerable population groups intersect with the smaller black and brown communities most easily accessible to researchers, individuals who had little to no say or knowledge of the true cost of their involvement as research participants. Examples of this range far and wide: the Holmesburg Prison experiments, Tuskegee Experiments, and MKUltra. Any and all methods of recruitment and experimentation, regardless of the long-term effects, were excusable as long as the scientific potential was promising.

Although copious ethical standards and guidelines have been put in place to ensure the ethical disgraces of the past are not repeated, “subject baiting” still takes place, if not more inventively: nowadays one may see the “make a quick buck” study ads pop up on street poles, in their local bulletin, or on their social media feeds, with little to no information on the specific parameters of the study that potential participants are being encouraged to volunteer for. Often, the first motive of the participating populations is the financial incentive with the only prerequisite being a willingness to have their body used and/or exploited.

Discrepancies in inclusivity can be examined in the everyday practices of research labs, as exemplified by the “representative sample” conundrum: Oftentimes, participant samples recruited for studies fall into the category of being W.E.I.R.D (White, Educated, Industrialized, Rich, and Democratic) (Henrich et al., 2010). While these populations are easy to recruit, especially on college campuses, they are not representative of the general population. One must ask if the notion of “ease of subject access” is a sufficient enough claim to not expand our research conventions in a way that would champion both inclusive and ethical science, a notion that is applicable in the case of both the populations we research and the research cohorts themselves. In comparison to other scientific fields, Neuroscience, especially on an academic level, is small. This means that the disparities in racial diversity are even more pronounced, as reflected in the aforementioned mere 6% minority tenure-track Neuroscience professors across academic institutions. The most challenging part lies in the fact that the only way in which this dynamic can shift is if opportunities for minorities to enter the Neuroscience field are made apparent, and if these diverse individuals see a place for themselves within this field. Statistics from 2011 revealed that only 12% of applications to Neuroscience graduate programs came from ethnic minorities, in comparison to the 18% applying to medical school and 23% to graduate school programs in general (2017, Reports, 2018), emphasizing that Neuroscience is still decades behind other fields in incorporating diversity into their research community.

## Conclusion

All in all, an examination of the dynamic between race and racial diversity within neuroscience will require a review of the historical treatment of minorities in the field. In order to fundamentally shift the field in the direction of equality, we have to focus efforts into making Neuroscience *equitable*—pursuing initiatives for the substantial amplification of diverse voices in the field until it becomes routine. Additionally, research practice can only progress when we consider that the work done thus far is still not sufficiently representative or universally appreciated. As long as we work in niches—“cultural neuroscience” instead of “neuroscience that reflexively integrates culture”—we remain limited as a field. If, however, the neuroscience community acknowledges its historical biases while reflecting upon current research practices and infrastructure, the field could become as dynamic, diverse, and ever-adapting as the very organ that it is dedicated to studying.

## Author Contributions

The author confirms being the sole contributor of this work and has approved it for publication.

### Conflict of Interest Statement

The author declares that the research was conducted in the absence of any commercial or financial relationships that could be construed as a potential conflict of interest.
